# A case of resected primary pulmonary pleomorphic carcinoma with long-term survival after multidisciplinary treatment

**DOI:** 10.1186/s40792-020-0794-3

**Published:** 2020-01-28

**Authors:** Yoshihito Iijima, Yuki Nakajima, Hiroyasu Kinoshita, Yasuyuki Kurihara, Yu Nishimura, Toshihiko Iizuka, Hirohiko Akiyama, Tomomi Hirata

**Affiliations:** 1 Division of Thoracic Surgery, Saitama Cancer Center, 780 Komuro, Ina-machi, Kita adachi-gun, Saitama, 362-0806 Japan; 2 Division of Pathology, Saitama Cancer Center, Saitama, Japan

**Keywords:** Lung cancer, Pleomorphic carcinoma, Multidisciplinary treatment, Long-term survival, Spontaneous regression

## Abstract

**Background:**

Generally, primary pulmonary pleomorphic carcinoma is resistant to treatment and has a poor prognosis. We report a case of resected primary pulmonary pleomorphic carcinoma with long-term survival after multidisciplinary treatment.

**Case presentation:**

A 74-year-old man with a history of emphysema, pneumoconiosis, and chronic bronchitis presented with left lung nodule and left adrenal tumor based on computed tomography. We suspected clinical T1bN0M1b, stage IVB lung cancer. Adrenalectomy of the left adrenal tumor yielded a definitive diagnosis of pleomorphic carcinoma. Chemotherapy was performed despite the spontaneous regression of lung lesions. Since lung lesions re-enlarged 11 months after adrenalectomy, the left lower lobe was partially resected followed by chemotherapy. The lung lesion was the primary lesion of the adrenal tumor. There was no recurrence 100 months after the lung resection.

**Conclusions:**

The patient experienced long-term survival after multidisciplinary treatment. Both multidisciplinary treatment and immunological mechanisms caused spontaneous regression of the primary lesion.

## Background

The histological type pulmonary pleomorphic carcinoma (PPC) was first proposed in the WHO classification in 1999. It was defined as a ‟poorly differentiated non-small cell carcinoma, namely squamous cell carcinoma, adenocarcinoma, or large cell carcinoma containing at least 10% or more spindle cells and/or giant cells, or a carcinoma consisting only of spindle or giant cell” [1]. Generally, primary pulmonary pleomorphic carcinoma is resistant to treatment and has a poor prognosis. We report a case of resected primary pulmonary pleomorphic carcinoma with long-term survival after multidisciplinary treatment.

## Case presentation

**Fig. 1:**
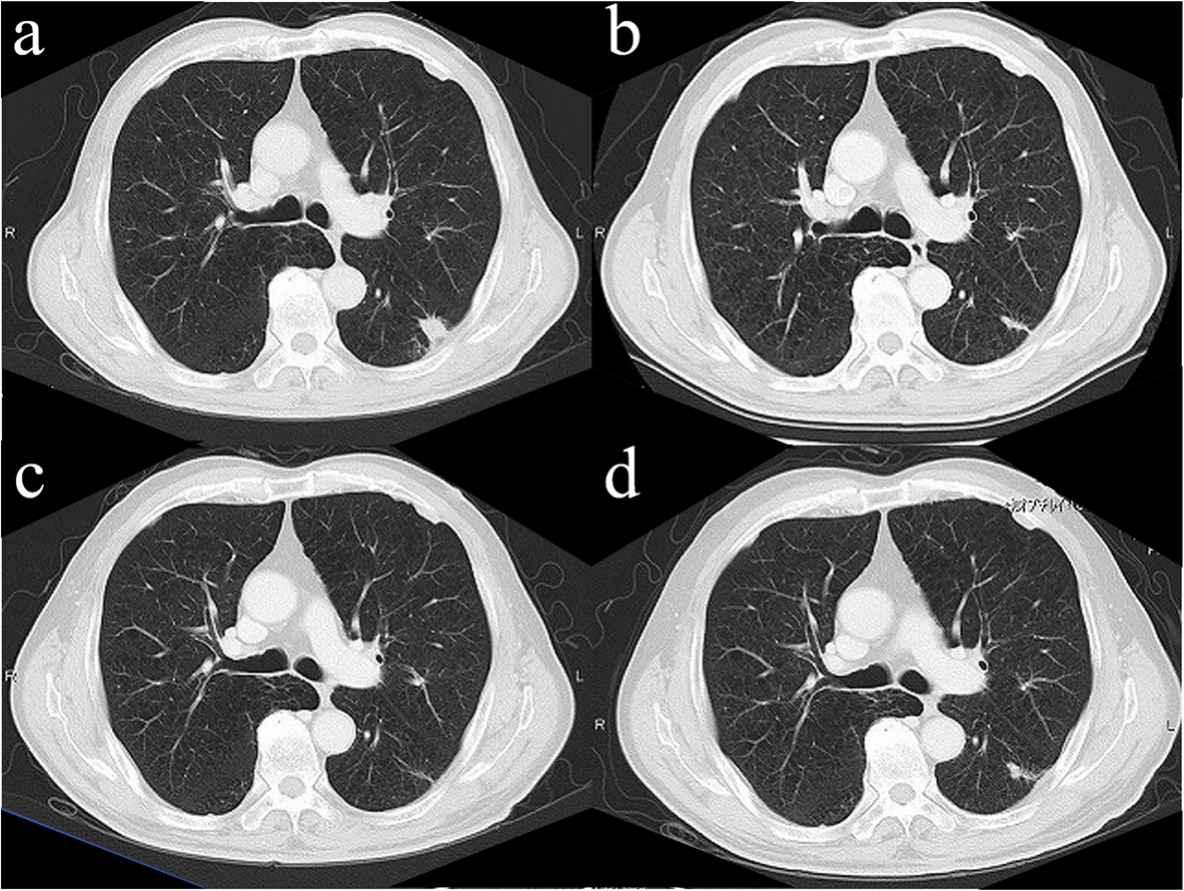
Computed tomography (CT). **a** Chest CT showed a well-circumscribed, 1.6-cm mass in the S6 segment of the left lower lobe. **b** CT performed before chemotherapy and 2 months after left adrenalectomy showed spontaneous regression of lung lesions. **c** After chemotherapy, the lung lesions became linear and nearly disappeared. **d** Lung lesions re-enlarged 11 months after adrenalectomy

**Fig. 2:**
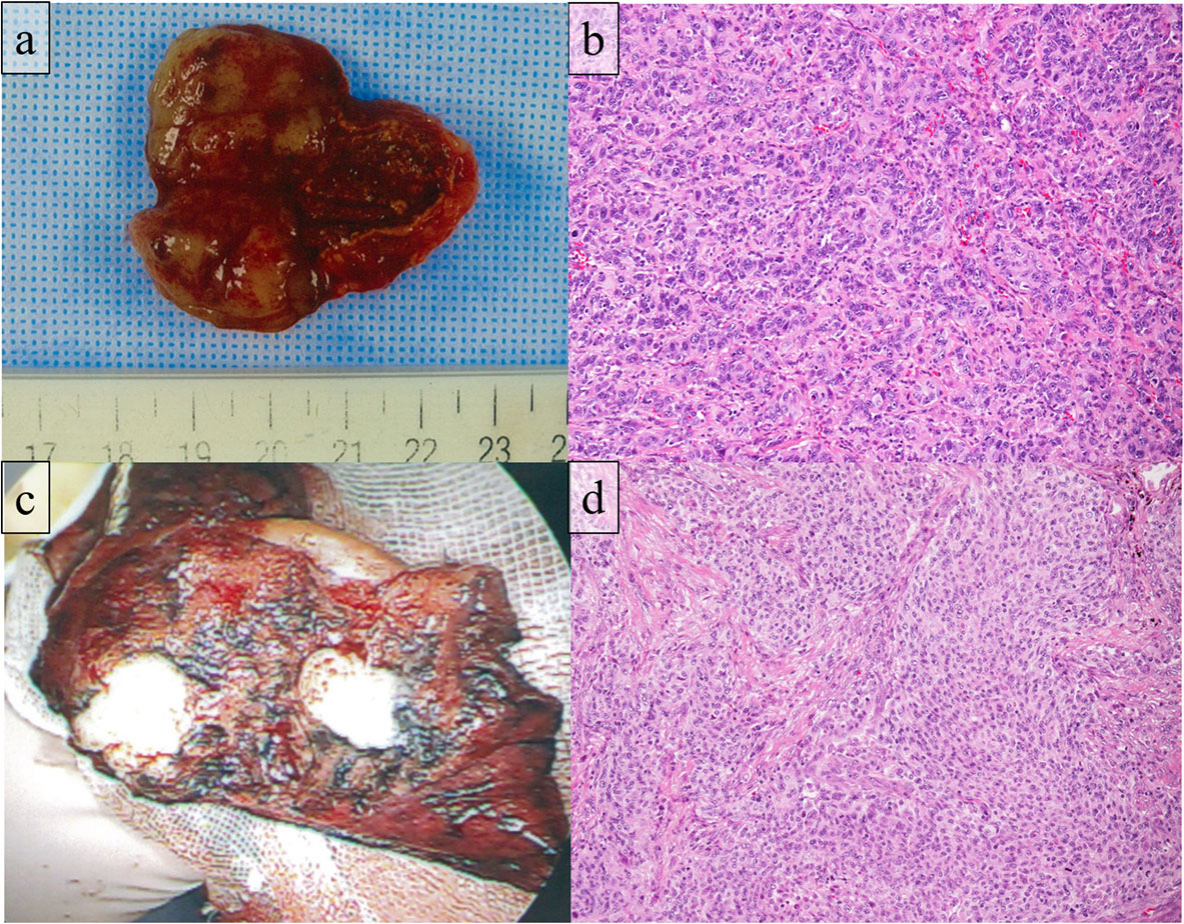
Pathological findings. **a** Macroscopic findings of the adrenal tumor: the tumor was a 3.5-cm gray-white tumor with a clear border. **b** Microscopic findings of the adrenal tumor: the tumor consisted of solid, proliferative, short, spindle cells without clear differentiation. **c** Macroscopic findings of the lung tumor: the tumor was a 1.4-cm, solid, white tumor with a clear border. **d** Microscopic findings of the lung tumor: there was no clear keratinization, inter-tissue bridge, or ductal structure, and the findings were similar to those of adrenal tumors

A 74-year-old man presented with a left lung nodule and left adrenal tumor based on computed tomography (CT) and was referred to us. He had a history of emphysema, pneumoconiosis, and chronic bronchitis. He had a history of smoking with a Brinkman index of 750. Serum tumor markers including carcinoembryonic antigen, cytokeratin 19 fragment, and pro-gastrin-releasing peptide were within the normal ranges. Chest and abdominal CT showed a well-circumscribed, 1.6-cm mass in the S6 segment of the left lower lobe (Fig. 1a) and a 3.5-cm mass in the left adrenal gland. Positron emission tomography (PET)-CT imaging revealed ^18^F-fluorodeoxyglucose (FDG) accumulation of a maximum standardized uptake value (SUV max) of 3.88 and 6.14 in the lung nodule and adrenal mass, respectively. We suspected clinical T1bN0M1b, stage IVB lung cancer of the left lower lobe and performed bronchoscopy. We performed left adrenalectomy to obtain a definitive diagnosis. Operation time and blood loss were 115 min and 56 ml, respectively. The left adrenal tumor was gray-white with a clear border of 3.5 cm consisting of solid, proliferative, short spindle cells without clear differentiation (Fig. 2a, b). Since a part of the tumor showed slight epithelial differentiation and most immunohistological epithelial markers were positive, metastatic cancer was considered. Immunohistochemical staining was positive for AE1/AE3, CAM5.2, epithelial membrane antigen (EMA), and vimentin but negative for thyroid transcription factor-1 (TTF-1), smooth muscle actin (SMA), desmin, and PE-10. Although it was TTF-1 negative and the lung lesion could not be determined as the primary lesion, there was a high possibility of metastasis of the primary PPC. CT after left adrenalectomy showed spontaneous regression of the lung lesion (Fig. 1b). Therefore, the possibility of inflammatory changes in S6 lung nodules could not be ruled out. Moreover, the possibility of cTXN0M1b, stage IVA lung cancer of unknown origin remained. Subsequently, chemotherapy with cisplatin and docetaxel was administered according to adjuvant chemotherapy of non-small cell carcinoma regimen. Finally, two courses of chemotherapy were administered. Chemotherapy was discontinued after two courses at the patient’s request due to nausea. After chemotherapy, the lung lesions became linear and nearly disappeared (Fig. 1c). Since lung lesions re-enlarged 11 months after adrenalectomy (Fig. 1d), PET-CT was performed that showed an FDG accumulation with SUV max of 2.72 in the lung nodule. Eventually, the pulmonary nodule was determined to be the primary lesion, and we decided to resect it for definitive diagnosis. In the respiratory function test immediately before the operation, both the forced expiratory volume in 1 s (1.24 l) and the forced expiratory volume 1 s percent (40.5%) were low. Due to high emphysema and poor lung function, we proposed a partial resection, and the patient agreed. Therefore, partial resection of the left lower lobe was performed. Operation time and blood loss were 60 min and 1 ml, respectively. Macroscopically, a solid white tumor with a clear border of 1.4 cm was observed (Fig. 2c). Histologically, there was no clear keratinization, inter-tissue bridge, or ductal structure, and the findings were similar to those of adrenal tumors (Fig. 2d). Immunohistochemical staining was positive for AE1/AE3, CAM5.2, EMA, and vimentin, and negative for TTF-1, SMA, desmin, and PE-10. The diagnosis was consistent with the primary lesions of adrenal tumors. Thus, three courses of chemotherapy with cisplatin and docetaxel were administered. There was no recurrence for 4 years and 3 months since the last treatment, and the patient was followed up at another hospital. After 8 years and 4 months since the lung resection, endocrine therapy was administered for the diagnosis of advanced prostate cancer. However, no recurrence of PPC was observed.

## Discussion

PPC is a relatively rare form of lung cancer with a poor prognosis. There are few reported cases, and therefore, its outcome and prognostic factors are not elucidated. Fishback et al. reported a 5-year overall survival rate of 10% [2], but in recent reports, the 5-year overall survival rate was approximately 33–80% [3–6]. There were various reports on prognostic factors, but there is no unified view. Most of the items examined were based on the patient background (gender, age, smoking history, etc.) and pathological findings (pathological stage, lymph node metastasis, tumor size, etc.) [2–8]. However, the prognostic factors for stage IV PPC are not clear. There are six resected cases of stage IV PPC including this case (Table 1) [9–12]. All cases had no lymph node metastasis (N0) or metastasis to a single organ (M1b), and thus, surgical resection was effective in these cases.

**Table 1:** Resected cases of stage IV pulmonary pleomorphic carcinoma

Case	Age	Sex	Primary site	Tumor diameter (cm)	Metastatic organ	Lymph node metastases	Chemotherapy	Regimen	Observation period after surgery (years)	Outcome	Reference
1	71	Male	RL	7	Brain	None	Pre	CBDCA+GEM	7	RF, A	[9]
2	48	Male	RU	6.5	Jejunum	None	None	–	6	RF, A	[10]
3	63	Male	LU	7.3	Ileum	None	Post	CBDCA+PTX	1	RF, A	[11]
4	69	Male	RU	4.8	Stomach	None	None	None	5	RF, A	[12]
5	62	Male	LU	3.3	Stomach	None	None	None	4	RF, A	[12]
6	74	Male	LL	1.4	Adrenal gland	–	Pre, post	CDDP+DOC	8.5	RF, A	Our case

*RL* right lower, *RU* right upper, *LU* left upper, *LL* left lower, *pre* preoperative lung resection, *post* postoperative lung resection, *CBDCA* carboplatin, *GEM* gemcitabine, *PTX* paclitaxel, *CDDP* cisplatin, *DOC* docetaxel, *RF* recurrence free, *A* alive

PPC is generally refractory to treatment, such as chemotherapy and radiotherapy, but the most effective treatment is surgical resection. There is no evidence-based regimen because it is a rare pathological type. Postoperative adjuvant chemotherapy is often administered with platinum combination therapy, as in the case of other non-small cell lung cancer [13]. In addition, new anticancer agents such as molecular targeted therapeutic agents and immune checkpoint inhibitors may be effective. Zhao et al. reported a significant correlation between high microvessel density in tumor tissue and high tumor reduction rate with bevacizumab combined chemotherapy in advanced-stage non-small cell lung cancer [14]. Bevacizumab combination therapy is expected to improve the therapeutic production phase. Moreover, the effective use of other molecular targeted therapeutic drugs, such as epithelial growth factor receptor (EGFR)-tyrosine kinase inhibitor (TKI) for cases with EGFR gene mutation [15], anaplastic lymphoma kinase (ALK)-TKI for ALK fusion gene-positive cases [16], or immunity checkpoint inhibitors (ICI) for cases with high expression of programmed cell death ligand 1 case in PPC [17, 18] is expected to improve the treatment strategy for PPC.

Several studies reported the spontaneous regression of tumors owing to an immune response triggered by CT-guided nodal biopsy, transbronchial lung biopsy, or surgical treatment [19–21]. In particular, the regression of tumors triggered by radiation therapy outside the irradiation field is called an abscopal effect [22], and the combined use of ICI and radiation therapy has attracted attention in recent years [23]. In this case, as well as in previous reports [20, 21], spontaneous regression of the primary lesion was observed after resection of the adrenal metastasis suggesting that some immunological mechanisms were involved in cancer control in addition to multidisciplinary treatment. We hope that immunological mechanisms for cancer control other than the abscopal effect will be elucidated in the future.

## Conclusions

The patient experienced long-term survival of the primary lung pleomorphic carcinoma. Both multidisciplinary treatment and immunological mechanisms caused spontaneous regression of the primary lesion of PPC.

## Data Availability

The patient data for this case report will not be shared to ensure patient confidentiality.

## Abbreviations

ALK: Anaplastic lymphoma kinase
CT: Computed tomography
EGFR: Epithelial growth factor receptor
EMA: Epithelial membrane antigen
FDG: ^18^F-Fluorodeoxyglucose
ICI: Immunity checkpoint inhibitors
PET: Positron emission tomography
PPC: Pulmonary pleomorphic carcinoma
SMA: Smooth muscle actin
SUV max: Maximum standardized uptake value
TKI: Tyrosine kinase inhibitor
TTF-1: Thyroid transcription factor-1
